# Supplementary material for: Flood hazard potential reveals global floodplain settlement patterns
Source: Nat Commun. 2023 May 16;14:2801. doi: 10.1038/s41467-023-38297-9 (PMC10188566; doi:10.1038/s41467-023-38297-9)
Supplement: Supplementary file 1 — Supplementary Information [file 41467_2023_38297_MOESM1_ESM.pdf]

Supplementary Materials for: Flood hazard potential reveals global floodplain settlement patterns

Laura Devitt, Jeffery Neal, Gemma Coxon, James Savage, Thorsten Wagener
*Corresponding author. Email: laura.devitt@bristol.ac.uk

Supplementary Text

Regional exposure on floodplains

The total number of people living on floodplains in Africa is 176 million – 92 million in North Africa, and 84 million in Sub-Saharan Africa. In North Africa, partially confined, confined and laterally unconfined floodplains account for 84%, 13% and 4% of all rivers reaches across the region. Whereas 27% of population exposure is found on laterally unconfined floodplains, and only 4% is found on confined floodplains. Egypt has the highest number of people living on floodplains in this region (63 million, approximately 63% it's total population) and 74% of this exposure is found on laterally unconfined floodplains, but only accounting for 26% of the floodplains on reaches in the country. In Sub-Saharan Africa, Nigeria has the largest number of people living on floodplains (21 million) and of this 16% are on laterally unconfined floodplains, while only making up 4% of the floodplains on all reaches. In the Middle East, 45 million people live on floodplains (17% of the total population across the region). Partially confined, confined, and laterally unconfined floodplains are found on 59%, 39% and 2% of all reaches in the region. However, 18% of population exposure is found on laterally unconfined floodplains, and only 11% is found on confined floodplains. Iraq and Iran have the largest numbers of people living on floodplains in the region (20 million and 18 million each). In Iraq, 55% of exposure is found on laterally unconfined floodplains, although only accounting for 6% of the reaches within the country.

In South and Central America, there are 97 million people settled on floodplains, which accounts for 15% of the total population across the region. Partially confined, confined, and laterally unconfined floodplains are found on 81%, 14% and 5% of all river reaches. Central America has the largest proportion of the population exposure found on confined floodplains (24%). Mexico has the largest population living on floodplains in this region (28 million people) and of this, 22% are living on confined floodplains.

In Europe, there are 98 million people settled on floodplains. Partially confined, confined and laterally unconfined floodplains are found on 83%, 13% and 5% of reaches across the region, however, 13% of population exposure is found on laterally unconfined floodplains, and only 3% is found on confined floodplains. Germany, Italy, and France have the highest total population living on floodplains, with 16 million, 14 million and 12 million people exposed, respectively.

In North America, 16% of the total population live on floodplains (59 million people), and of this, 55 million are in the United States (17% of the population are exposed to flooding). In the US, partially confined, confined and laterally unconfined floodplains are found on 79%, 17% and 4% of all river reaches, however, 11% of the population exposure is found on laterally unconfined floodplains, and only 5% is found on confined floodplains.

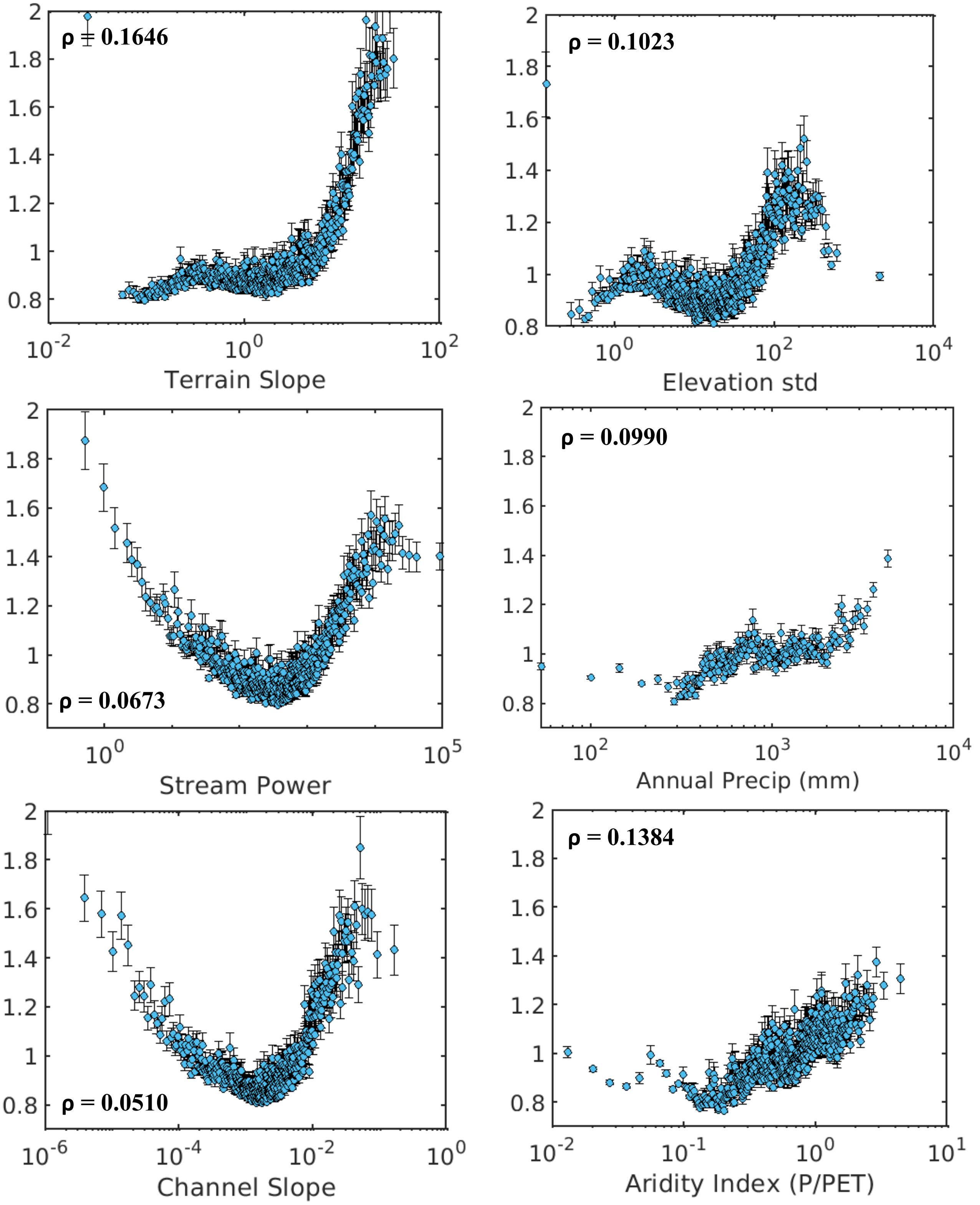

Fig. S1.

Correlation between physical attributes and river reach sensitivity to flooding. Physical properties have been calculated for each river reach, and their influence on the sensitivity of the river reach (b_r) to changing flood magnitude is assessed. Reaches are put into 500 bins (2000 points per bin) each representing 0.2% of the total data. Local topography is the dominant control. Spearman Rank Correlation Coefficients are given for the unbinned data. The error bars display the standard error of the mean for each bin.

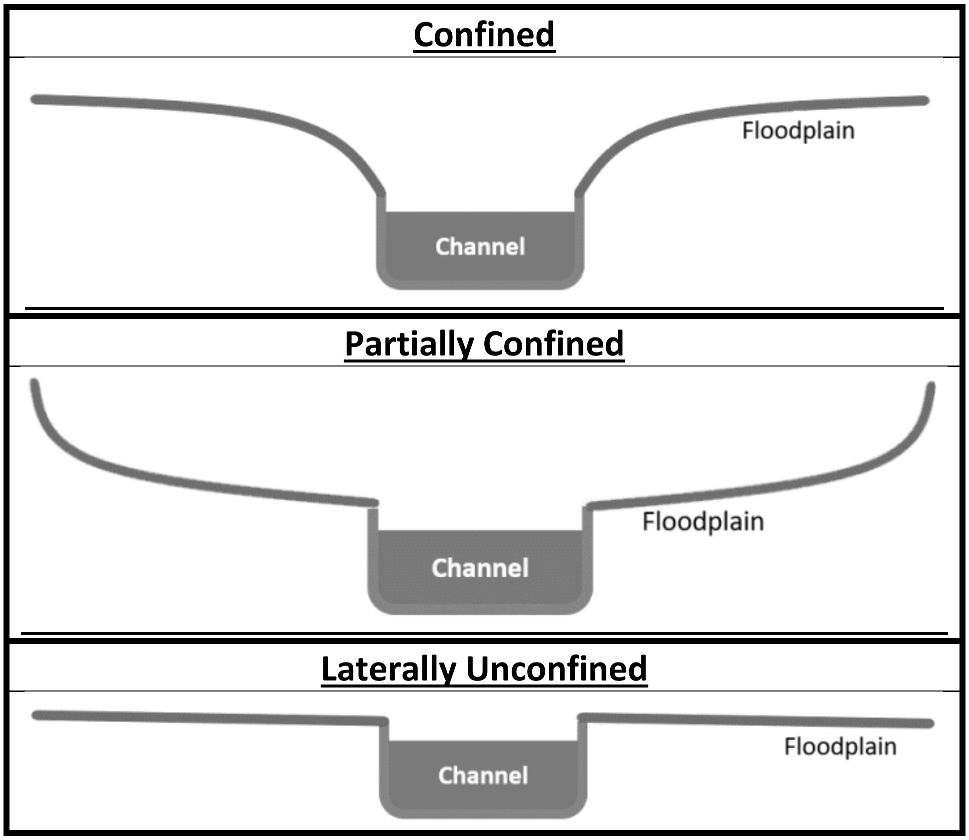

Fig. S2.

Conceptual diagrams of the three main types of floodplains identified. Confined floodplains are found on steep bedrock streams in mountainous areas. Flood extents grow most rapidly during rare, extreme magnitude events due to steep slopes next to the river channel constraining how flood extents can grow laterally during frequent, low magnitude events. Partially confined are the dominant type of floodplain across the globe and are found on transitional streams, e.g., valley bottoms. Flood extents grow most rapidly during frequent, low magnitude events, which is due to the wide and flat terrain next to the river channel that meets a break in slope which can constrain the growth of inundation. Laterally unconfined floodplains are very wide and flat and are found on low slope alluvial streams. They are unbounded which allows for exponential growth in inundation area. Flood extents grow most rapidly during rare, extreme magnitude events.

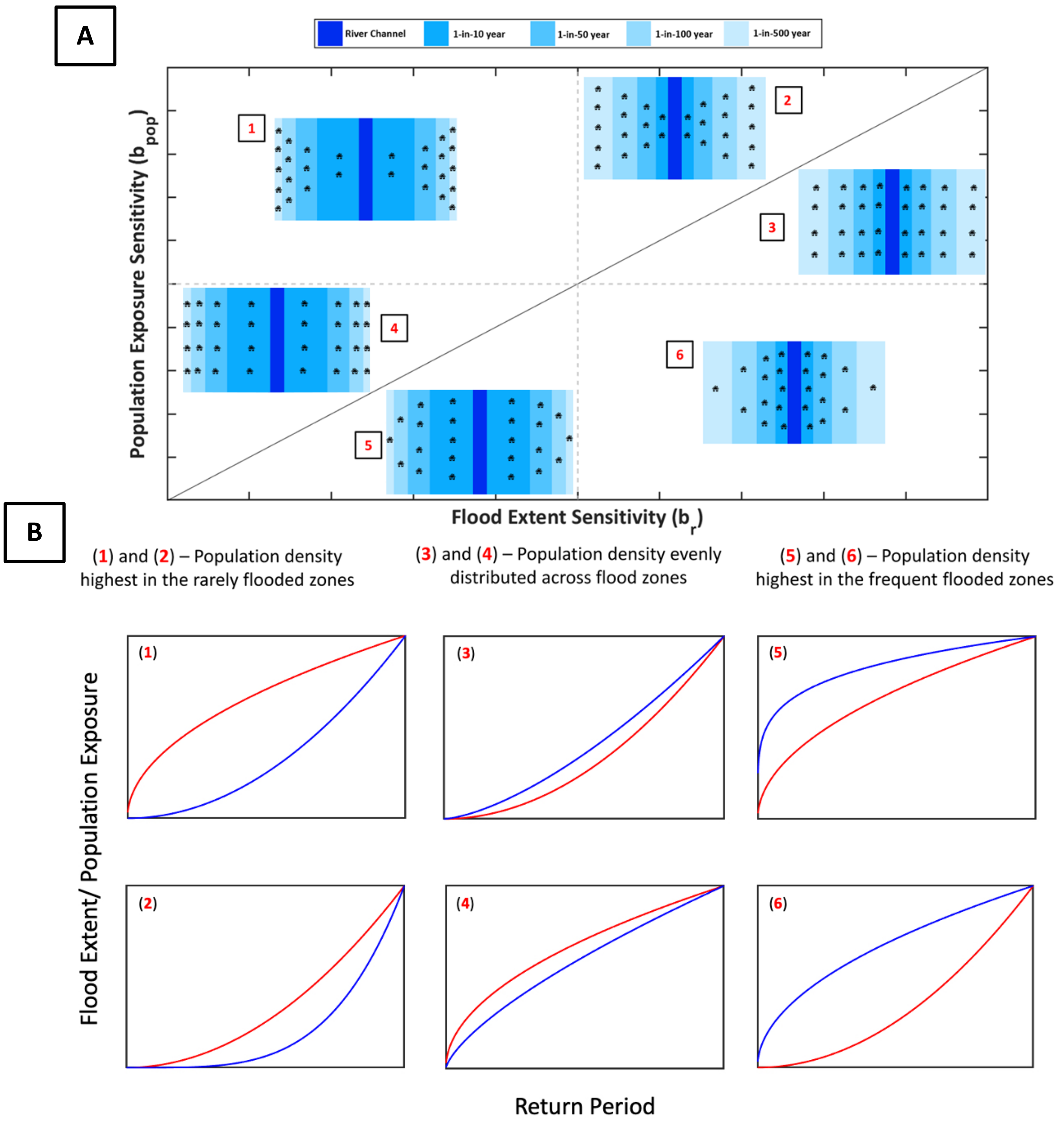

Fig. S3.

Diagram of how physical flood hazard and population exposure sensitivity interact. A) Simplified diagram to demonstrate the sensitivity of flooded extent and population exposed to increasing return period. The flooded extent of the different return periods is shown in blue. Population settled in different zones of the floodplain is represented by the house icon. On the left side of the vertical grey dashed line, flooded extents grow most rapidly during frequent, low magnitude events. On the right side of the vertical grey dashed line, flooded extents grow most rapidly during rare, extreme magnitude events. The diagrams have been added to the sections of the plot that corresponds with Figure 3 (main text) and Figure S4 (supplement) as a conceptual representation of how the sensitivity parameters of the flooded extents and population exposure interact. This provides us with an indication of where the highest density

of population exposure is in relation to the different flood hazard zones. **B)** Example growth curves for flood extent and population exposure that correspond with the sections of the plot.

Note: The scale in (A) differs from that in Figure 3 & S4, where we have used a log scale when plotting the data. This is due to the values that b_r and b_{pop} take. When b is less than 1, values range between 0 and 1, however, when b is greater than 1, theoretically values can range between 1 and infinity.

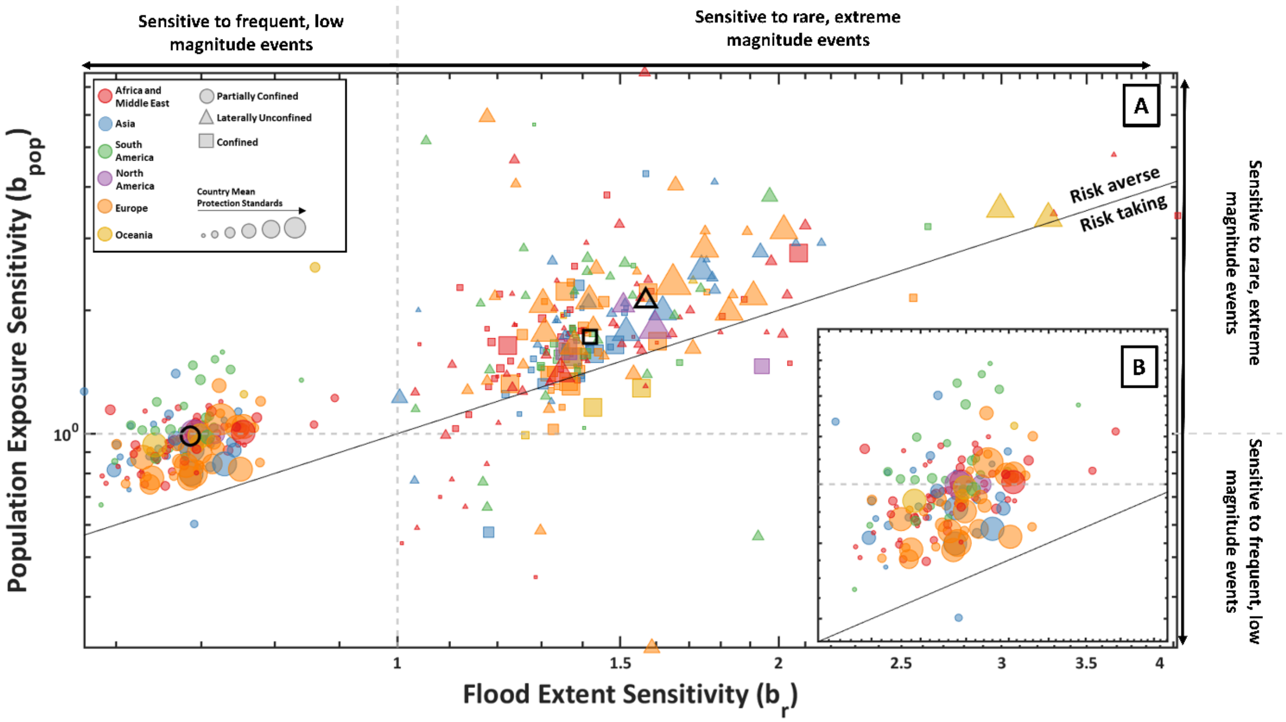

Fig. S4.

Interaction between flood hazard and population exposure sensitivity considering mean country level standard of flood protection. A) The country level mean flood hazard and population exposure sensitivity parameters (b_r & b_{pop}) are calculated for each floodplain type. Each country has three marker shapes – one each for the mean sensitivity parameters on confined, partially confined, and laterally unconfined floodplains. The grey dashed lines indicate where the flood hazard (x axis) and population exposure (y axis) growth curves transition from growing most rapidly during frequent, low magnitude events to during rare, extreme magnitude events. Markers closest to the 1:1 line have growth curves with similar shapes for flood extent and population, which indicates that population is distributed evenly across the floodplain. Locations above the 1:1 line have greater population densities on rarely inundated areas of the natural floodplain, while locations below the line have greater population densities in frequently flooded areas. Where b_{pop} < 1 more people live on the frequently flooded floodplain, while b_{pop} > 1 indicates more people live in rarely flooded areas. (e.g., on laterally confined floodplains many countries have higher population densities in rarely flooded areas but overall greater population in frequently flooded areas). Point shapes represent the floodplain category for each country, the sizes have been scaled based on the mean standard of protection in each country, and the colour refers to the region/continent that the country is in. The bold open shapes represent the centroid of all the data points for each of the floodplain types. Note: The standard of protection level is a country-wide mean value extracted from the FLOPROS dataset and is not floodplain type specific. **B)** Close view of the partially confined floodplain results. Here we see that many of the European countries that are between the 1:1 line and the horizontal grey dashed line (i.e. where population in absolute terms is great in frequently flooded areas of the floodplain) have high standards of structural protection. Continent specific plots are included in Fig. S5 for ease of visualisation.

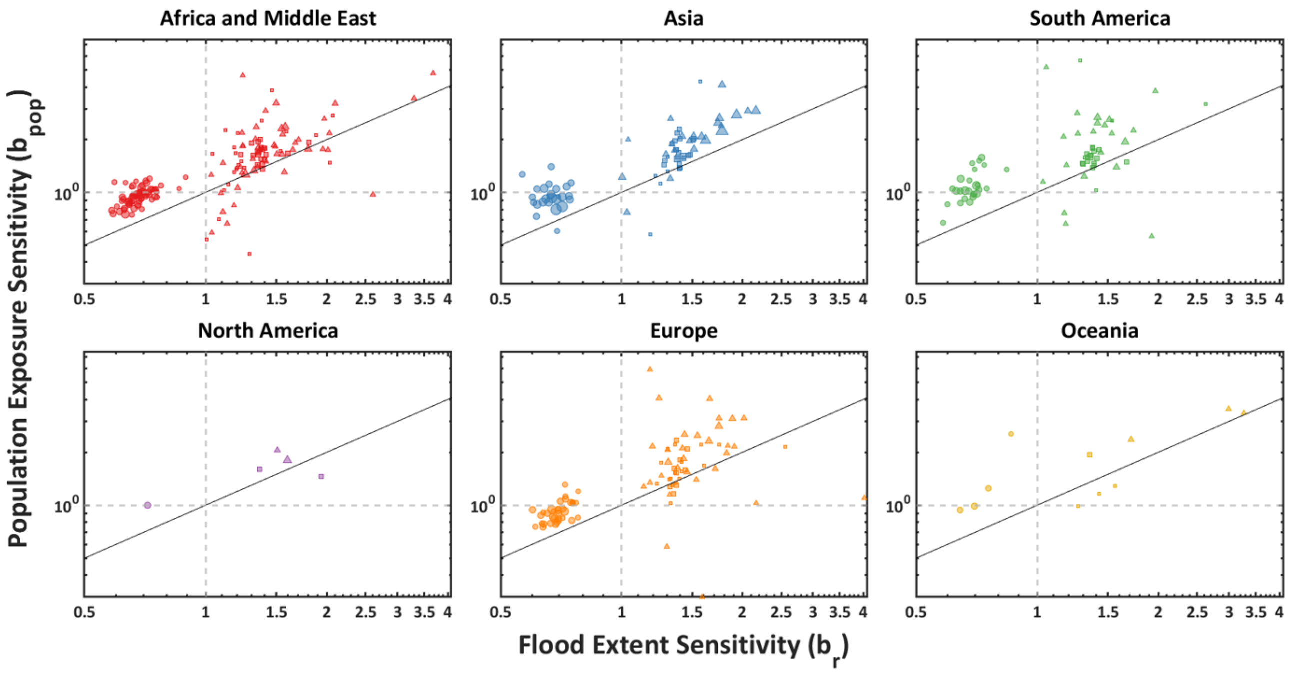

Fig. S5.

Interaction between flood hazard and population exposure sensitivity by continent. Country level mean flood extent and population exposure sensitivity parameters. The regions presented in Figure 3 (main text) and Figure S4 have been separated in order to highlight the regional differences. Point shapes represent the floodplain category for each country (circles for partially confined, triangles for laterally unconfined, squares for confined), the sizes have been scaled based on the number of people living on the different floodplain types in each country, and the colour refers to the region/continent that the country is in.

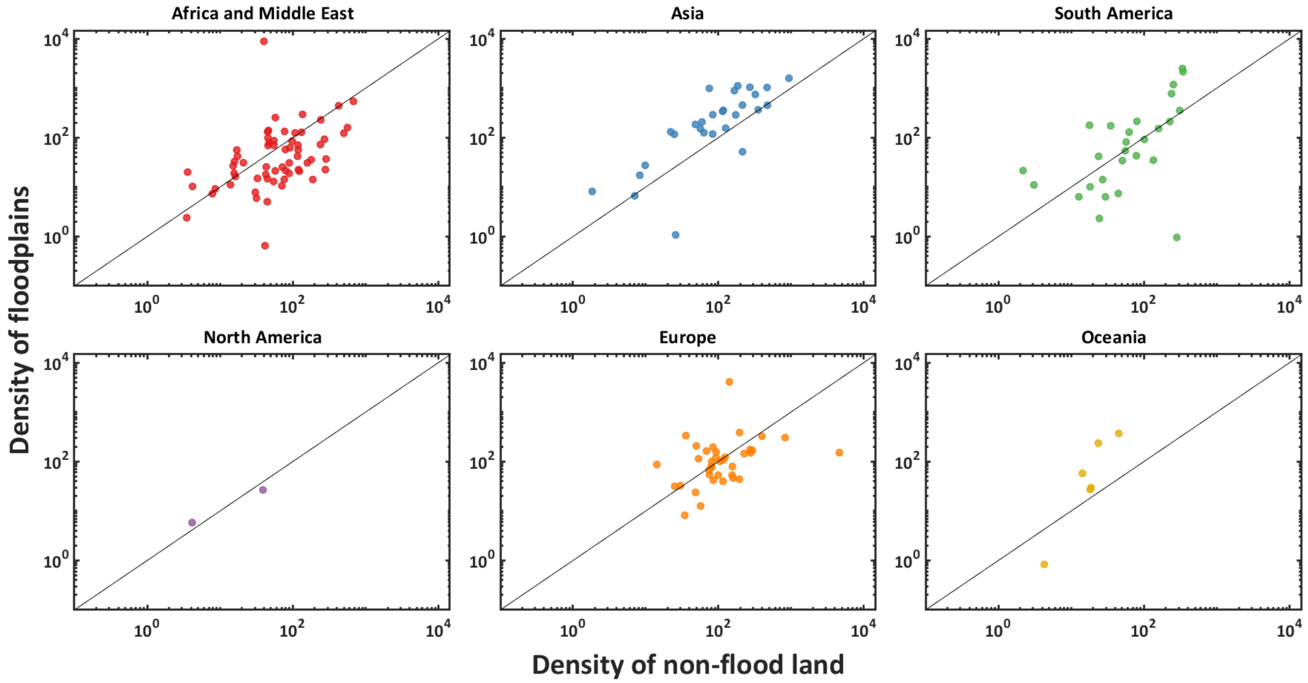

Fig. S6

Population density per km^2 of floodplain and non-floodplain land. Floodplain land is defined as the 1-in-1000-year flood extent. Non-flood land is defined as everything that is beyond this. Here, we have compared the population density on floodplains vs. the non-floodplain land. Points on the 1:1 line represent where the density of population per km^2 is the same on the floodplain and outside of this area. Points below the 1:1 line represent where the population density is higher on the non-floodplain land. Points above the 1:1 line represent where the population density is higher on the floodplain.

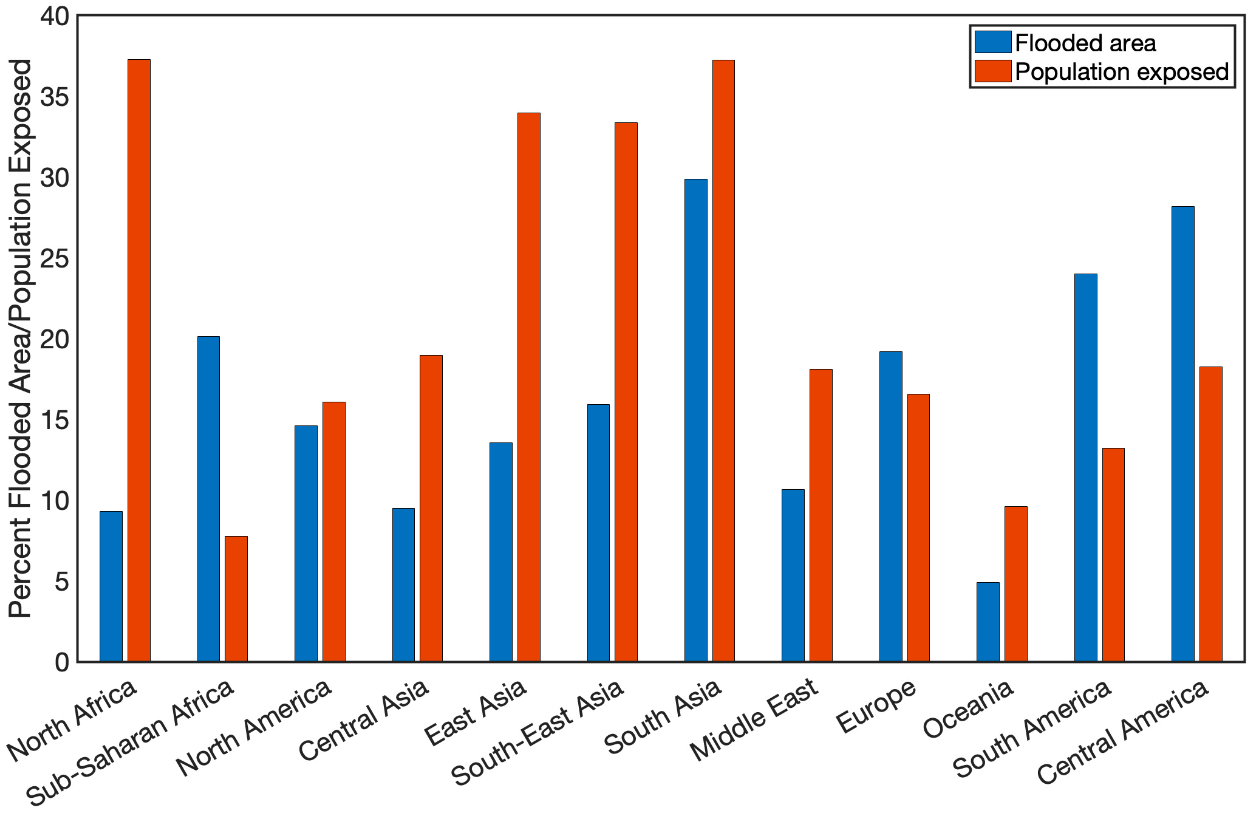

Fig. S7

Regional total floodplain area and population exposure. The floodplain area as a percentage of the total land area, and the population exposure as a percentage of the total for each region. There are clear regional differences in where floodplains are being preferentially settled and developed.

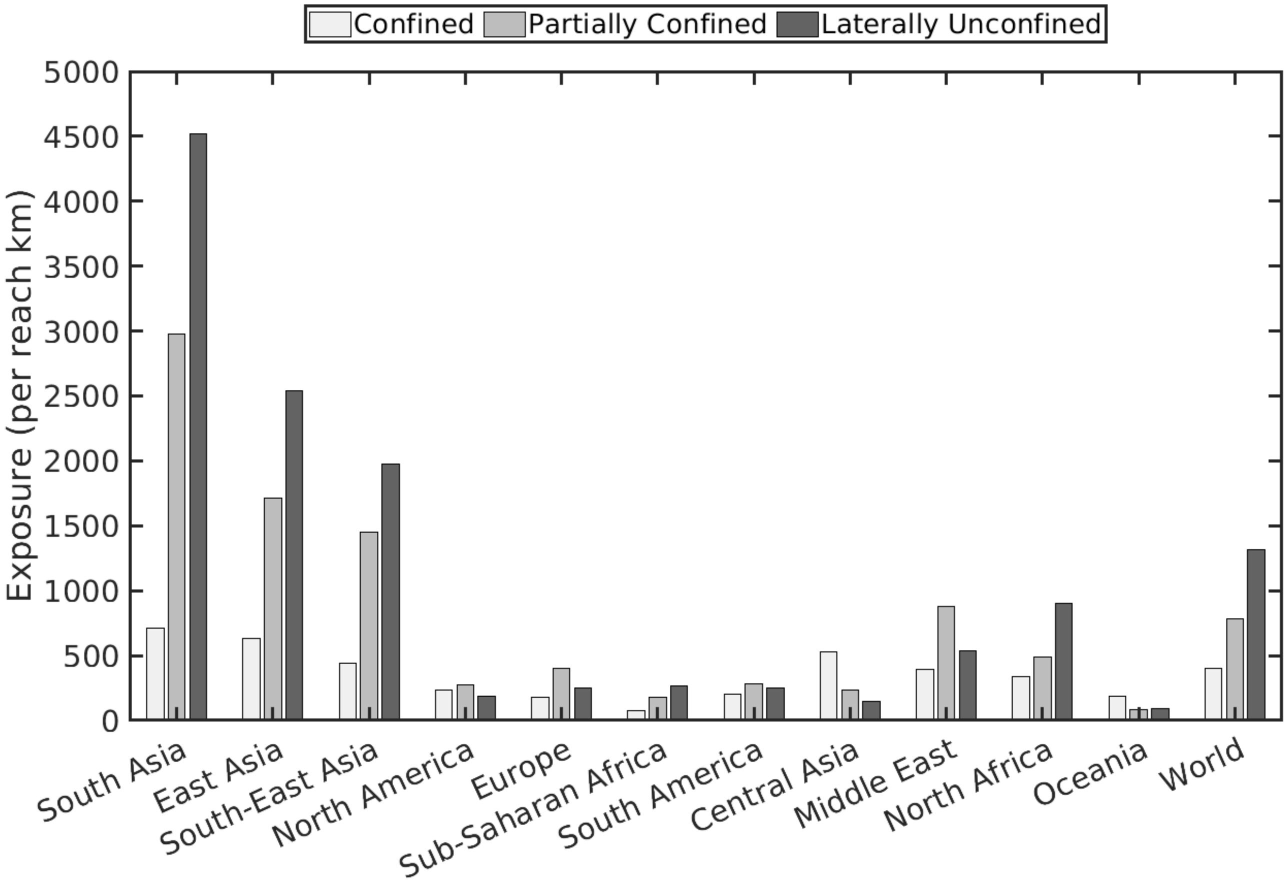

Fig. S8.

Exposure density of the floodplain categories. River reaches have been separated into their sub-region and the floodplain category. Exposure density has been calculated as the number of people who are exposed per kilometre of river reach in each of the floodplain categories. This highlights the high density of exposure that is found on floodplains in Asia.

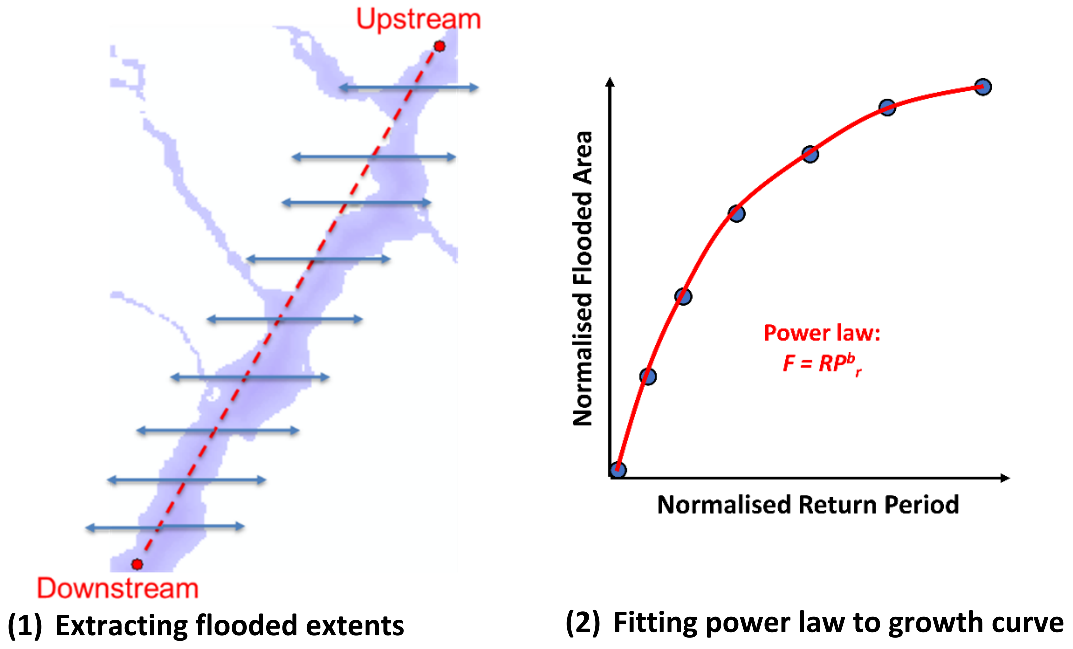

Fig. S9.

Diagram of quantifying the sensitivity of flooded extents. 1) Upstream inflow points and downstream boundary conditions are used to define reaches along the global river network. Flooded extents are extracted between these upstream and downstream points along a diagonal transect using the reach length as an effective search radius. This is done for each return period. 2) extracted flooded areas are normalised and plotted against the normalised return period. A power law is fitted to these points. The exponent parameter of this power law is used to describe the shape of the growth curve of the flooded extents.
